# Histone deacetylase 3 facilitates TNFα-mediated NF-κB activation through suppressing CTSB induced RIP1 degradation and is required for host defense against bacterial infection

**DOI:** 10.1186/s13578-022-00814-6

**Published:** 2022-06-03

**Authors:** Liping Yang, Shengchuan Chen, Jingyan Xia, Ying Zhou, Linan Peng, Huimin Fan, Yu Han, Lihua Duan, Genhong Cheng, Heng Yang, Feng Xu

**Affiliations:** 1 Department of Infectious Diseases, The Second Affiliated Hospital, Zhejiang University School of Medicine, Hangzhou, 310009 China; 2 Institute of Systems Medicine, Chinese Academy of Medical Sciences & Peking Union Medical College, Beijing, 100005 China; 3 Suzhou Institute of Systems Medicine, Suzhou, 215123 China; 4 Department of Gastrointestinal Surgery, The First Affiliated Hospital, Zhejiang University School of Medicine, Hangzhou, 310009 China; 5 Department of Radiation Oncology, The Second Affiliated Hospital, Zhejiang University School of Medicine, Hangzhou, 310009 China; 6 Department of Obstetrics and Gynecology, The First Affiliated Hospital of Soochow University, Suzhou, 215123 China; 7 Department of Rheumatology and Clinical Immunology, Jiangxi Provincial People’s Hospital, The First Affiliated Hospital of Nanchang Medical College, Nanchang, 330000 China; 8 Department of Microbiology, Immunology and Molecular Genetics, University of California, Los Angeles, CA 90095 USA

**Keywords:** HDAC3, CTSB, RIP1, NF-κB

## Abstract

**Background:**

As important enzymes regulating acetylation, histone deacetylases (HDACs) participate in a series of cell physiological process. However, the mechanisms responsible for individual HDAC family members in regulating innate immunity remained to be elucidated. Here we sought to reveal the mechanism of HDAC3 in regulating the inflammatory response of macrophages.

**Methods:**

RNAseq was done to detect the transcriptional influence of HDAC3 on macrophages. Kyoto Encyclopedia of Genes and Genomes was used to reveal the change of signaling pathways after HDAC3 knockout. CHIPseq was done to detect the deacetylation modification of HDAC3 on chromosome. Western blot, immunofluorescence, and real-time quantitative PCR were used to measure the change of genes and proteins’ levels. Mice were intratracheal instillation with lipopolysaccharide or *Pseudomonas aeruginosa* to determine the influence of HDAC3 on inflammatory response in vivo.

**Results:**

HDAC3-deficient macrophages had increased expression of cathepsins resulting from elevated histone acetylation. Over-expressed cathepsins such as cathepsin B (CTSB) caused remarkable degradation of receptor (TNFRSF)-interacting serine-threonine kinase 1 (RIP1), which reduced TNFα mediated NF-κB activation and inflammatory response. Consistently, mice with macrophage specific knockout of HDAC3 were impaired in inflammatory response and thereby susceptible to *Pseudomonas aeruginosa* infection.

**Conclusion:**

HDAC3 was required for protecting RIP1 from degrading by CTSB in macrophages. Decreased RIP1 in HDAC3 knockout macrophages impaired TNFα mediated NF-κB activation. Our studies uncovered important roles of HDAC3 in the regulation of cathepsin-mediated lysosomal degradation and RIP1-mediated inflammatory response in macrophages as well as in host defense against bacterial infection.

**Supplementary Information:**

The online version contains supplementary material available at 10.1186/s13578-022-00814-6.

## Introduction

As important deacetylases, the function of HDACs has been well studied [1–3]. Through interacting with histones and non-histones, they exert various effects on chromatin remodeling, transcriptional regulation and protein stability [4], with important contributions to physiological or pathological processes. Eighteen HDAC family members of eukaryotes are divided into four classes. Class I (HDAC1, HDAC2, HDAC3, HDAC8), Class II (HDAC4, HDAC5, HDAC6, HDAC7, HDAC9, HDAC10) and Class IV (HDAC11) HDACs are Zn^++^-dependent enzymes, while Class III (Sirt1-Sirt7) HDACs are NAD^+^-dependent enzymes [5]. The importance of HDACs promotes the development of HDAC inhibitors, five of which have been approved by US Food and Drug Administration (FDA) or China FDA (CFDA) for the treatment of hematological tumors [6]. The influence of HDACs on macrophages has also been taken notice recently by studying both repression and activation of gene transcription in mouse bone-marrow-derived macrophages (BMDM) with myeloid-specific loss of HDAC3 [7]. However, the mechanisms responsible for individual HDAC family members in regulating inflammatory response in macrophages and in host defense against bacterial infections remained to be elucidated.

Lysosomal degradation system is the major intracellular degradation system. Both extracellular materials and cellular components can be delivered to the lysosome for degradation [8]. Cathepsins are a family of proteases [9], acting as important executors of lysosomal degradation system through digesting of internalizes wasted cell proteins and peptides. Most of the cathepsin members belong to cysteine proteases, and a few are aspartic protease (CTSD, CTSE) and serine protease (CTSA, CTSG). Abnormal expression of cathepsins can lead to the disorder of lysosomal degradation system’s activity, inducing the unbalance of protein degradation and cell homeostasis. A series of articles have been reported the links between cathepsins and diseases including cancer [10], mental illness [11], etc.

RIP1 is an essential molecule in TNFα or Myd88-independent Toll-like receptor (TLR) activation induced NF-κB activity. Reduced NF-κB activity and inflammatory response was observed in cells with RIP1 genetic interference [12, 13] or drug inhibition [14]. In accordance with these, in vivo application with Nec-1s, a RIP1 inhibitor showed decreased inflammatory response after injection with LPS [15]. However, impaired inflammatory response can sensitize host to infection. RIP1 deficient mice showed more death after infection with *Yersinia* [16, 17] or *Candida albicans* [18], indicating the key role of RIP1in host defense against bacterial and fungus infections.

Here we reported that HDAC3 was a critical transcriptional repressor of cathepsins. Elevated expression of cathepsins in HDAC3 deficient macrophages results in remarkable lysosomal degradation of RIP1, leading to reduced TNFα mediated NF-kB activation and the inflammatory response. More importantly, macrophage specific HDAC3 knockout mice were impaired in host defense against bacterial infection. These results indicated a novel function of HDAC3 in host defense against bacterial infection by suppressing cathepsin-mediated RIP1 degradation and also raised a potential concern of opportunistic bacterial infections during clinical application of HDAC inhibitors.

## Results

### Elevated expression of cathepsins in HDAC3 deficient macrophages

**Fig. 1:**
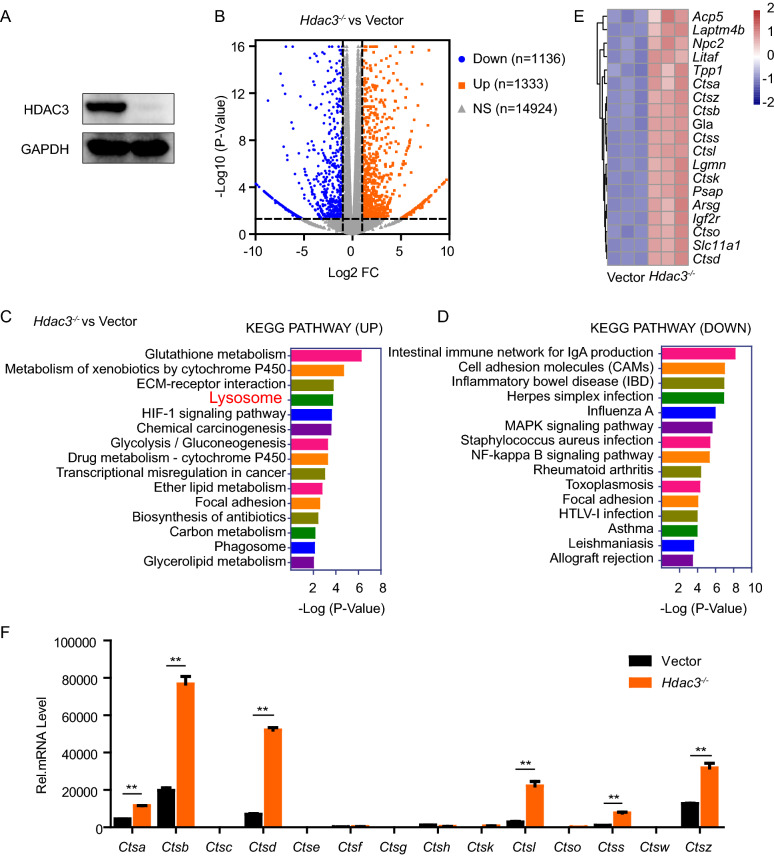
Elevated expression of cathepsins in HDAC3 deficient macrophages. **A** Western blot analysis of HDAC3 in Vector and *Hdac3*^*−/*−^ RAW264.7 cells. **B** Volcano plot for genes significantly changed (≥ 2 fold change, P < 0.05) after HDAC3 knockout. **C**, **D** Kyoto Encyclopedia of Genes and Genomes (KEGG) analysis for genes significantly changed (≥ 2 fold change, P < 0.05) after HDAC3 knockout. **E** Heat map of relative lysosome related mRNA expression. **F** PCR analysis of *Ctsa*, *Ctsb*, *Ctsc*, *Ctsd*, *Ctse*, *Ctsf*, *Ctsg*, *Ctsh*, *Ctsk*, *Ctsl*, *Ctso*, *Ctss*, *Ctsw*, *Ctsz* in Vector and *Hdac3*^*−/*−^ RAW264.7 cells. Data are representative of three independent experiments and showed as mean ± SEM. **P < 0.01

In order to explore the role of HDAC3 in the regulation of the cellular activity of macrophages, we constructed HDAC3 knockout murine monocytic cell line RAW264.7 cells through the CRISPR/Cas9 gene editing system (Fig. 1A). RNAseq was done to detect the change after HDAC3 knockout. Results showed 1333 genes and 1136 genes were significantly upregulated or downregulated after HDAC3 knockout, respectively (Fig. 1B). KEGG analysis of genes significantly changed after HDAC3 knockout showed that HDAC3 deficiency in macrophages significantly upregulated genes involved in the lysosomal pathway (Fig. 1C, D). We surprisingly found that the mRNA levels of multiple cathepsins which are important executors for protein degradation of lysosomal degradation system mentioned above, including *Ctsa*, *Ctsb*, *Ctsd*, *Ctsl*, *Ctss* and *Ctsz*, were significantly elevated in HDAC3 knockout macrophage cells by RNAseq analysis (Fig. 1E). These changes were confirmed by RT-qPCR (Fig. 1F).

**Fig. 2:**
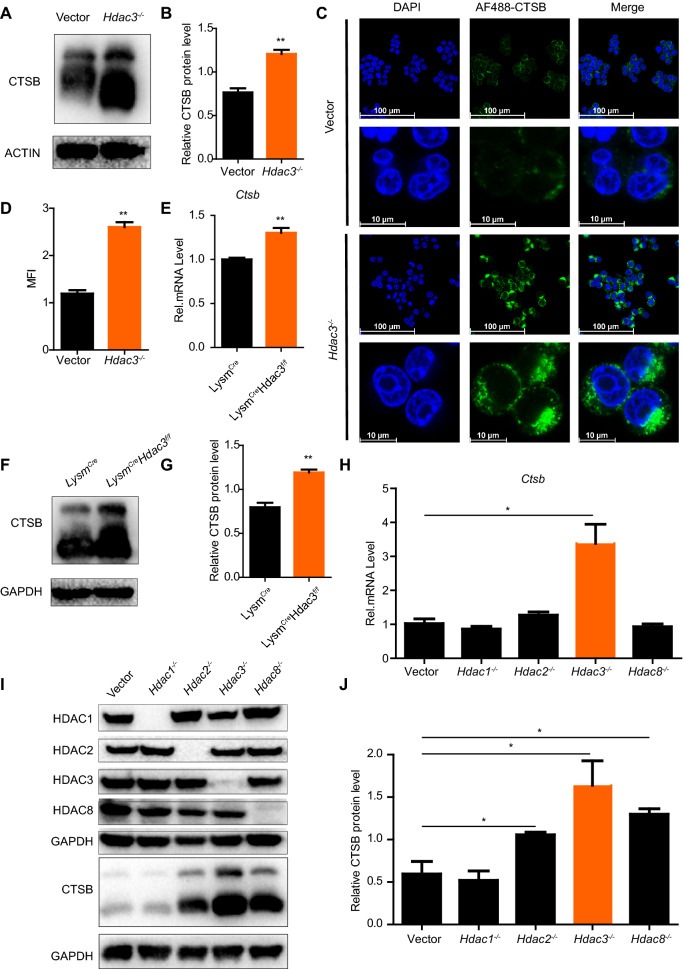
HDAC3 deficient macrophages have elevated expression of *Ctsb*. **A**, **B** Western blot analysis of CTSB in control and HDAC3 deficient RAW264.7. **C**, **D** Confocal micrographs of RAW264.7 cells stained with DAPI (blue, DNA) and antibodies against CTSB (AF488). Scale bar: 100/10 μm. **E** PCR analysis of *Ctsb* in *Lysm*^*Cre*^ and *Lysm*^*Cre*^*Hdac3*^*f/f*^ BMDMs. **F**, **G** Western blot analysis of CTSB in *Lysm*^*Cre*^ and *Lysm*^*Cre*^*Hdac3*^*f/f*^ BMDMs. **H** PCR analysis of *Ctsb* in Vector, *Hdac1*^*−/−*^, *Hdac2*^*−/−*^, *Hdac3*^*−/−*^, *Hdac8*^*−/−*^ RAW264.7 cells. **I**, **J** Western blot analysis of HDAC1, HDAC2, HDAC3, HDAC8 and CTSB in Vector, *Hdac1*^*−/−*^, *Hdac2*^*−/−*^, *Hdac3*^*−/−*^, *Hdac8*^*−/−*^ RAW264.7 cells. Data are representative of three independent experiments and showed as mean ± SEM. *P < 0.05 and **P < 0.01

CTSB, a critical protein in the lysosomal system, was among the cathepsins with the highest expression in macrophages (Fig. 1F). In consistent with mRNA expression, the protein levels of CTSB detected by western blot and confocal analysis were also significantly higher in *Hdac3*^*−/*−^ as compared to Vector RAW264.7 cells (Fig. 2A–D). We have further generated BMDMs from *Lysm*^*Cre*^ and *Lysm*^*Cre*^*Hdac3*^*f/f*^ mice. The successful loss of HDAC3 in *Lysm*^*Cre*^*Hdac3*^*f/f*^ BMDMs was confirmed by PCR (Additional file 1: Fig. S1A) and western blot analysis (Additional file 1: Fig. S1B). And we found that HDAC3-deficient BMDMs had higher levels of CTSB mRNA and protein than *Lysm*^*Cre*^ BMDMs (Fig. 2E–G). We also explored the influence of other members of Class I HDACs on CTSB with HDAC1, HDAC2, HDAC8 knockout RAW264.7 cells generated by CRISPR/Cas9. Besides HDAC3, which had the most obvious effect on CTSB, HDAC2 and HDAC8 knockout cells also showed slightly elevated CTSB expression, while HDAC1 had no influence on the expression of CTSB (Fig. 2H–J). We also observed among Class I HDACs, CTSD was expressed at the highest levels in *Hdac3*^*−/−*^ RAW264.7 (Additional file 1: Fig. S2A–C). Taken together, these data indicated the important role of HDAC3 in suppressing the expression of CTSB.

### HDAC3-dependent histone deacetylation on the promoters of cathepsins

**Fig. 3:**
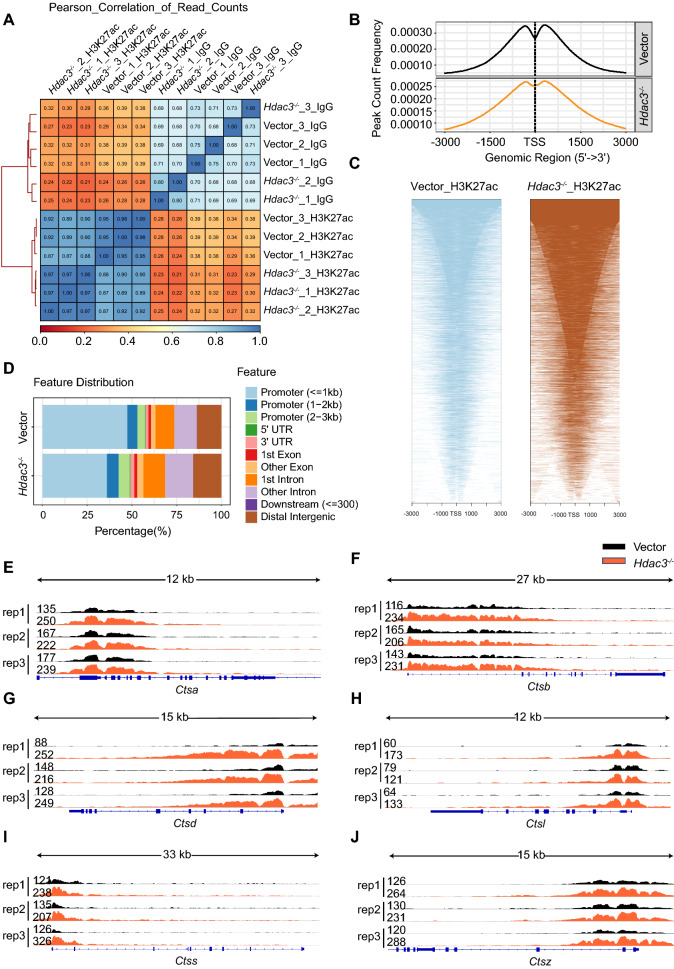
HDAC3 deacetylases the histones on the promoters of cathepsins. **A** Heat map representation of correlation between each biological replicates of Vector and *Hdac3*^*−/−*^ RAW264.7 cells for H3K27ac. **B** Distribution of H3K27ac-binding loci relative to transcriptional start site (TSS). **C** H3K27ac signals in Vector and *Hdac3*^*−/−*^ RAW264.7 at TSS. **D** Genome-wide distribution of H3K27ac ChIP-seq peaks in RAW264.7 cells. **E**–**J** The Integrative Genomics Viewers (IGVs) for H3K27ac occupied on the promoter of *Ctsa*, *Ctsb*, *Ctsd*, *Ctsl*, *Ctss* and *Ctsz*. Each CHIPseq data of histone marks were merged from three individual replicates

As histone deacetylation has negative effects on transcription and HDAC3 is an important deacetylase, we speculated that HDAC3 knockout might promote the histone acetylation on the promoter of these cathepsins to increase their expression. Confocal analysis revealed that HDAC3 was mainly located in the nucleus (Additional file 1: Fig. S3). Chromatin immunoprecipitation (ChIP) was then performed against H3K27ac. Correlation matrix between ChIP-seq experiments demonstrated good consistency between samples (Fig. 3A). The CHIP peak-calling program Model Based Analysis for CHIP-Seq data (MACS2) revealed that H3K27ac located near the transcriptional start sites (TSS) (Fig. 3B, C) and its binding was enriched on the promoter of the genes (Fig. 3D), indicating the regulation of acetylation on the transcription. More importantly, we observed significantly increased acetylation on the promoter of *Ctsa*, *Ctsb*, *Ctsd*, *Ctsl*, *Ctss*, *Ctsz* in HDAC3-deficient macrophages as compared to the corresponding Vector macrophages (Fig. 3E–J). These data indicated that HDAC3 may negatively regulated the expression of these cathepsins through deacetylase the histones on their promoters.

### Reduced protein level of RIP1 in HDAC3 deficient macrophage

**Fig. 4:**
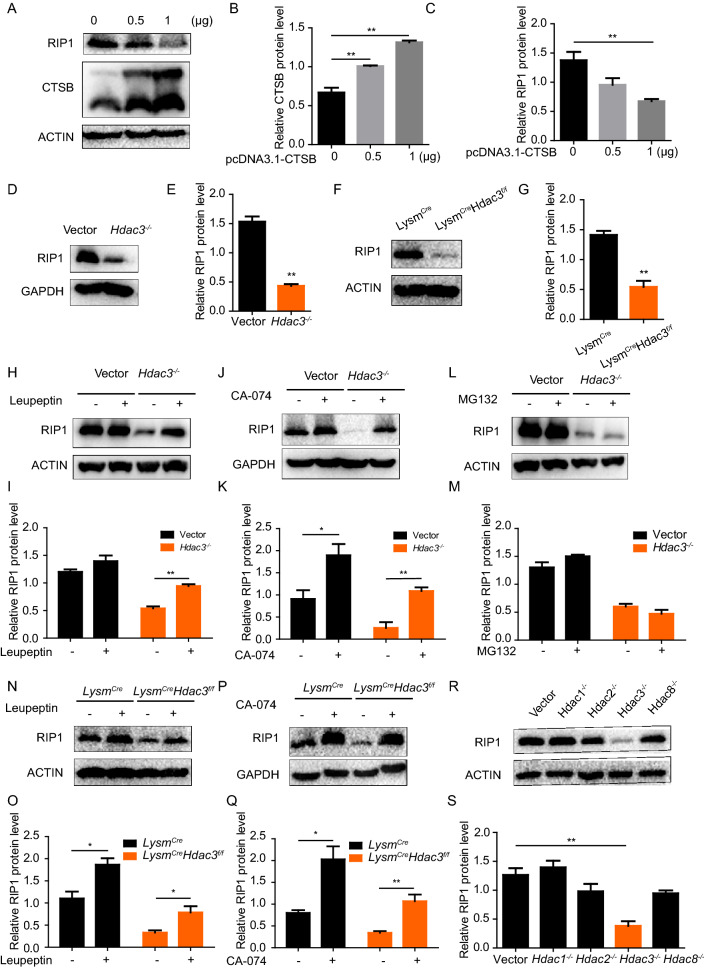
Reduced protein level of RIP1 is observed in HDAC3 deficient macrophage. **A**–**C** Western blot analysis of RIP1 in Neuro-2a cells transfected with CTSB. **D**–**G** Western blot analysis of RIP1 in control and HDAC3 deificient RAW264.7 and BMDMs. **H**–**M** Western blot analysis of RIP1 in Vector and *Hdac3*^*−/−*^ RAW264.7 stimulated with Leupeptin (25 μM), CA-074 (10 μM) or MG132 (10 μM) for 12 h. **N**–**Q** Western blot analysis of RIP1 in *Lysm*^*Cre*^ and *Lysm*^*Cre*^*Hdac3*^*f/f*^ BMDMs stimulated with Leupeptin (100 μM) or CA-074 (40 μM) for 12 h. **R**, **S** Western blot analysis of RIP1 in Vector, *Hdac1*^*−/−*^, *Hdac2*^*−/−*^, *Hdac3*^*−/−*^, *Hdac8*^*−/−*^ RAW264.7 cells. Data are representative of three independent experiments and showed as mean ± SEM. *P < 0.05 and **P < 0.01

CTSB has been reported to be involved in the cleavage of RIP1 [19], which is a critical molecule on the signaling pathway for TNFα mediated NF-κB activation [20, 21]. We therefore explored the influence of the elevated CTSB expression in HDAC3 knockout macrophages on RIP1 expression. Decreased expression level of RIP1 protein was observed in Neuro-2a cells transfected with CTSB (Fig. 4A–C) and also in HDAC3 deficient RAW264.7 and BMDMs as compared with the corresponding control cells (Fig. 4D–G). Furthermore, application of either Leupeptin, an inhibitor of the lysosomal system or CA-074, an inhibitor of CTSB, restored the protein level of RIP1 in HDAC3 deficient RAW264.7 cells (Fig. 4H–K). The same phenomenon was also observed in HDAC3 deficient BMDMs (Fig. 4N–Q). As CTSB degrades proteins mainly in lysosomes, we also observed the co-localization of RIP1 and lysosomes in Neuro-2a cells. Cells treated with CA-074 had increased the resident of RIP1 in lysosomes as compared to control cells, indicating CTSB mediated RIP1 degradation mainly occurred in lysosomes (Additional file 1: Fig. S4). Lysosomal system and ubiquitin–proteasome are two main ways involved in protein degradation. We also applied cells with MG132, an inhibitor of the proteasome mediated degradation system and found it failed to restore the protein level of RIP1 in HDAC3 knockout macrophages (Fig. 4L, M). The above results indicated that the decreased protein level of RIP1 was caused by the over-reactive lysosomal degradation after HDAC3 knockout. We also explored the protein level of RIP1 in Vector, *Hdac1*^*−/−*^, *Hdac2*^*−/−*^, *Hdac3*^*−/−*^, *Hdac8*^*−/−*^ RAW264.7 cells and found HDAC3 deficiency in macrophages resulted in the most significantly decreased protein level of RIP1, which was consistent with the increase of CTSB protein levels (Fig. 4R, S).

### Impaired RIP1-mediated NF-κB signaling activity in HDAC3 deficient macrophages

**Fig. 5:**
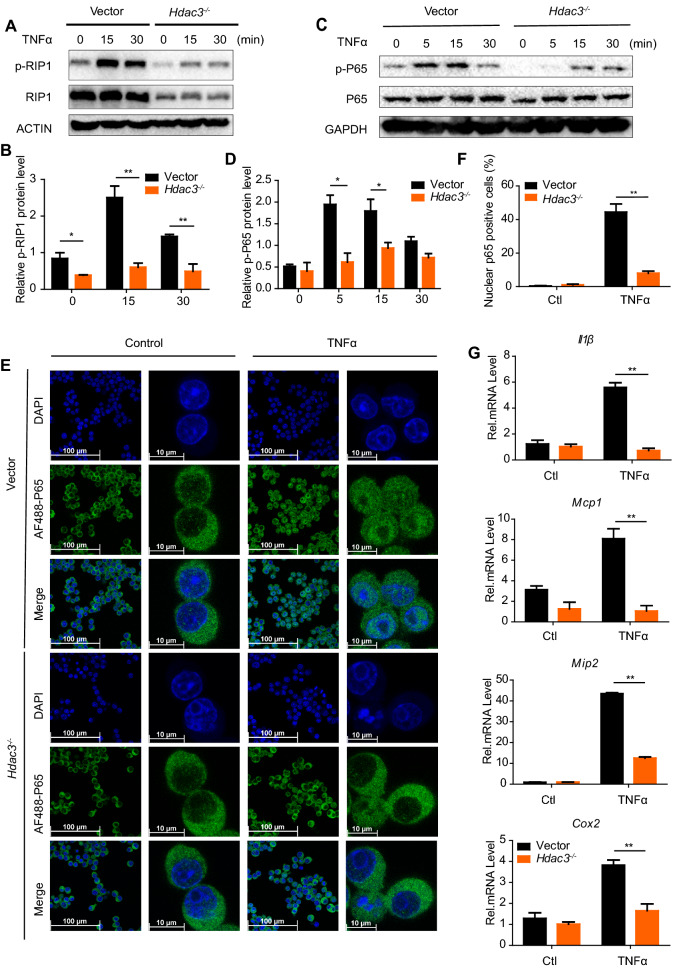
HDAC3 deficiency in macrophages results in impaired RIP1-dependent NF-κB signaling activity. **A**, **B** Western blot analysis of phosphorylated and total RIP1 in Vector and *Hdac3*^*−/−*^ RAW264.7 stimulated with TNFα (20 ng/ml) for indicated time. **C**, **D** Western blot analysis of phosphorylated and total P65 in control and HDAC3 deficient RAW264.7 stimulated with TNFα (20 ng/ml) for indicated time. **E**, **F** Confocal micrographs of RAW264.7 stimulated or unstimulated with TNFα (20 ng/ml) for 15 min stained with DAPI (blue, DNA) and antibodies against P65 (AF488). The nuclear P65 positive cells were calculated. Scale bar: 100/10 μm. Ctl, Control. **G** PCR analysis of *Il1β*, *Mcp1*, *Mip2* and *Cox2* in Vector and *Hdac3*^*−/−*^ RAW264.7 stimulated with TNFα (20 ng/ml) for 1 h. Data are representative of three independent experiments and showed as mean ± SEM; *P < 0.05 and **P < 0.01

RIP1 is a critical upstream molecule for the activation of NF-κB signaling pathway after TNFα stimulation which functions as docking sites for NF-κB essential modulator (NEMO), TGFβ-activated kinase 1 (TAK1) and the TAK1-binding proteins (TAB1 and TAB2) [22]. Our studies showed that Vector and *Hdac3*^*−/−*^ RAW264.7 cells were stimulated with TNFα, both the total and phosphorylated RIP1 levels were significantly lower in *Hdac3*^*−/−*^ as compared to Vector RAW264.7 cells (Fig. 5A, B). Consistent with this, the impaired activation of downstream NF-κB signaling pathway was observed in *Hdac3*^*−/−*^ RAW264.7 after TNFα stimulation (Fig. 5C, D). And the nuclear localization of P65 after TNFα stimulation was remarkably decreased in *Hdac3*^*−/−*^ RAW264.7 (Fig. 5E, F), indicating HDAC3 was required for the RIP1 mediated NF-κB activation. We also performed PCR analysis for the mRNA expression of TNFα inducible cytokines and chemokines and observed the significantly decreased expression of *IL1β*, *Mcp1, Mip2* and *Cox2* in *Hdac3*^*−/−*^ RAW264.7 cells (Fig. 5G)*.* These data emphasized the importance of HDAC3 in RIP1-NF-κB induced inflammatory response.

### The role of HDAC3 in host defense against *pseudomonas aeruginosa* infection

**Fig. 6:**
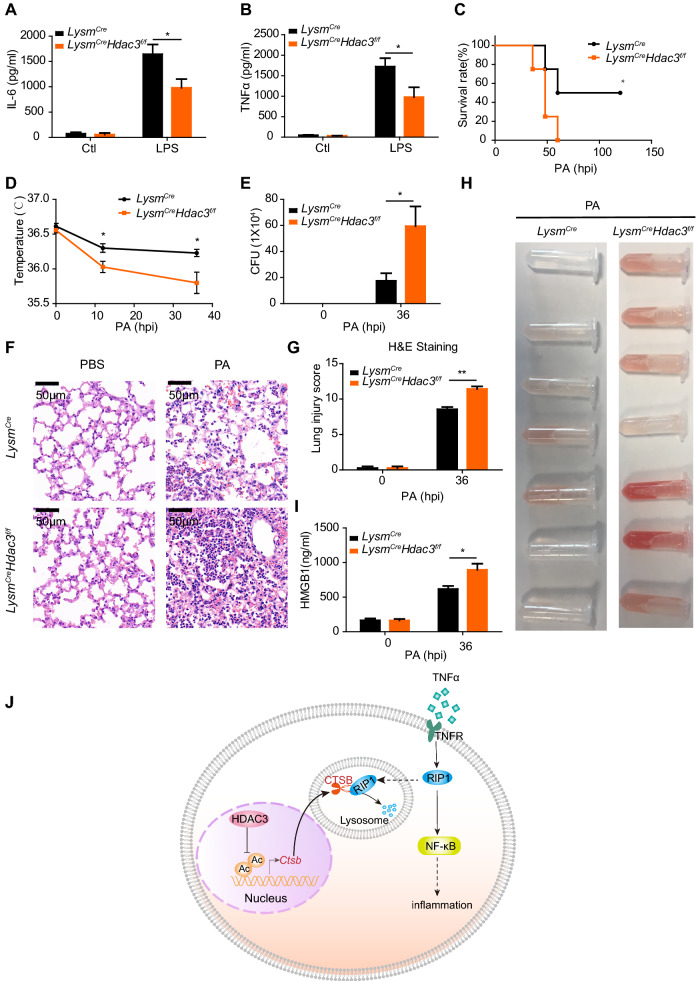
HDAC3 deficiency in macrophages aggravates pseudomonas aeruginosa induced acute lung injury due to impaired inflammatory response. **A**, **B** ELISA analysis of IL-6 and TNFα in BALF of *Lysm*^*Cre*^ and *Lysm*^*Cre*^*Hdac3*^*f/f*^ mice uninstilled or intratracheally instilled with LPS (5 mg/kg) (12 h). **C** Survival curve of 8-week-olds *Lysm*^*Cre*^ and *Lysm*^*Cre*^*Hdac3*^*f/f*^ mice intratracheally received with *pseudomonas aeruginosa* [Colony forming unit (CFU) = 2 × 10^7^]. PA, *Pseudomonas aeruginosa*. **D** Temperature of *Lysm*^*Cre*^ and *Lysm*^*Cre*^*Hdac3*^*f/f*^ mice intratracheally received with *pseudomonas aeruginosa* (CFU = 2 × 10^7^) monitored for 36 h. **E** CFU of *pseudomonas aeruginosa* in BALF of *Lysm*^*Cre*^ and *Lysm*^*Cre*^*Hdac3*^*f/f*^ mice uninstilled or intratracheally instilled with *pseudomonas aeruginosa* (CFU = 2 × 10^7^) for 36 h was calculated. **F**, **G** H&E staining of *Lysm*^*Cre*^ and *Lysm*^*Cre*^*Hdac3*^*f/f*^ mice intratracheal unreceived or received with *pseudomonas aeruginosa* (CFU = 2 × 10^7^) for 36 h and the lung injury score was calculated. Scale bar: 50 μm. **H** Pulmonary hemorrhage of *Lysm*^*Cre*^ and *Lysm*^*Cre*^*Hdac3*^*f/f*^ mice received with *pseudomonas aeruginosa* (CFU = 2 × 10^7^) for 36 h was judged by the color of BALF. **I** ELISA analysis of HMGB1 in the BALF of *Lysm*^*Cre*^ and *Lysm*^*Cre*^*Hdac3*^*f/f*^ mice uninstilled or intratracheally instilled with *pseudomonas aeruginosa* (CFU = 2 × 10^7^) for 36 h. **J** Schematic diagram of how HDAC3 regulates RIP1 mediated inflammatory response of macrophages. Data are showed as mean ± SEM; n = 4–9 mice per group; *P < 0.05 and **P < 0.01

To further validate the effect of HDAC3 on inflammatory response of macrophages in vivo, *Lysm*^*Cre*^ and *Lysm*^*Cre*^*Hdac3*^*f/f*^ mice were intratracheally instilled with endotoxin (LPS). The BALF was collected for the detection of the inflammatory cytokine secretion. Decreased secretion of IL-6 and TNFα was found in *Lysm*^*Cre*^*Hdac3*^*f/f*^ mice (Fig. 6A, B), indicating the impaired inflammatory response in vivo. Insufficient inflammatory response lowers the body’s resistance to infection. We further conducted lung infection experiments in *Lysm*^*Cre*^ and *Lysm*^*Cre*^*Hdac3*^*f/f*^ mice by intratracheal drip of *pseudomonas aeruginosa*. Consistent with our expectations, the survival rate of *Lysm*^*Cre*^*Hdac3*^*f/f*^ was significantly lower than *Lysm*^*Cre*^ mice (Fig. 6C). The basic status of *Lysm*^*Cre*^*Hdac3*^*f/f*^ mice were also significantly worse, manifested by a remarkable reduced activity, lower body temperature (Fig. 6D) and difficulty in breathing. In addition, higher numbers of bacteria were accumulated in *Pseudomonas aeruginosa* infected *Lysm*^*Cre*^*Hdac3*^*f/f*^ than *Lysm*^*Cre*^ mice (Fig. 6E). Further pulmonary histopathological examination showed that *Pseudomonas aeruginosa* infection led to more severe lung injury, manifested as pulmonary edema, hemorrhage, inflammatory cell infiltration, and cellulose exudation, in *Lysm*^*Cre*^*Hdac3*^*f/f*^ than *Lysm*^*Cre*^ mice (Fig. 6F, G). Increased hemorrhage was also showed as the color of the BALF of *Lysm*^*Cre*^*Hdac3*^*f/f*^ was redder than *Lysm*^*Cre*^ mice after *Pseudomonas aeruginosa* infection (Fig. 6H). HMGB1 is a conserved nuclear protein in cells. It is loosely bound to chromosomes and can be passively secreted through the broken cell membrane in necrotic cells. Thus, it can be an indicator of lung injury. We observed that the levels of HMGB1 in *Lysm*^*Cre*^*Hdac3*^*f/f*^ mice were higher than those in *Lysm*^*Cre*^ mice after infection (Fig. 6I). In conclusion, HDAC3 was required for inhibiting the expression of *Ctsb* through possible deacetylating the histone on its promoter. Degradation of RIP1 resulted from elevated expression of *Ctsb* in HDAC3 deficient macrophages impaired TNFα mediated NF-κB activation and eventually impaired the inflammatory response and anti-infection immunity of host (Fig. 6J).

## Discussion

As the major protein post-translational modification, acetylation which depends on the balance between HAT and HDAC determines the normal development, structure and function of cells. Abnormal expression or mutations of HDAC family members are associated with numerous diseases including cancers and autoimmune diseases. As more and more HDAC inhibitors have been and are being developed, potential unknown targets and side effects of these HDAC inhibitors should be considered given the broad physiological and pathology functions of this family members. Our studies here have provided evidence demonstrating that HDAC3 is an important regulator in suppressing molecule lysosomal degradation system, and facilitating inflammatory response. HDAC3 knockout resulted in overexpressed CTSB through promoting acetylation of the histones on its promoters. The increased CTSB acted as a scissor for the degradation of RIP1, leading to the impaired activation of TNFα-induced NF-κB signaling pathway. More importantly, mice with macrophage specific knockout of HDAC3 showed more susceptible than control mice to *pseudomonas aeruginosa*, suggesting the critical role of HDAC3 in host defense against bacterial infection.

HDACs may affect the lysosomal degradation system through multiple steps, including the influence on the lysosomal pH, the expression of lysosome related protein, autophagosome-lysosome fusion, lysosomal exocytosis and the acetylation modification of targeted protein. Reduced lysosomal pH was observed in neck squamous cell carcinoma cell line applied with HDAC inhibitor trichostatin A, leading to increased cathepsin activity and cell death [23]. Another HDAC inhibitor SAHA was able to increase the transcriptional activity of TFEB [24] and the expression of LAMP1, LAMP2, CTSB [25]. HDAC10 was required for depressing LAMP2A expression [26]. The deacetylation of syntaxin 17 (STX17), a key autophagosomal SNARE protein mediating autophagosome maturation by HDAC2 [27] and Cortactin by HDAC6 [28] promotes autophagosome-lysosome fusion. As for lysosomal exocytosis, HDAC inhibitor ITF2357 was able to induce hyperacetylation of tubulin, preventing the exocytosis of IL-1β-containing secretory lysosomes [29]. In addition, the acetylation modification of targeted protein such as PKM [30] and Tau [31] promoted themselves degraded by lysosomal system. However, these previous studies are mainly based on the effects of HDAC inhibitors on particular targets, the role of specific HDAC in overall lysosomal degradation system is not clear. Here we first reported that HDAC3 was a key regulator in suppressing the expression of multiple cathepsin genes through deacetylating histones on their promoter regions. Moreover, our RNAseq analysis between control and HDAC3 knockout macrophages have identified many more HDAC3 suppressed genes in the lysosomal degradation system. Further studies especially proteomic studies are needed to understanding the broad impact of HDAC3 in controlling lysosomal degradation system and potential side effects of using HDAC inhibitors.

Inflammatory response as an important part of immune system involves regulation of numerous proinflammatory genes such as TNF-α, IL-1β and IL-6 and anti-inflammatory genes such as IL-10, IL-4 and TGF-β. Defective inflammatory response may lead to uncontrolled infectious diseases and cancers whereas over reactive inflammatory response may be associated with various inflammatory diseases. Although many different signal transduction pathways are involved in regulating inflammatory response, the NF-kB pathways are known to play critical roles. Previous studies have reported that the CTSB inhibitor CA-074 decreased NF-κB activity and inflammatory factors’ expression [32] and CTSB knockdown inhibited doxorubicin-induced NF-κB activation [33], suggesting an important role of CTSB for inflammation. However, the molecular mechanisms responsible for regulation of inflammatory response by HDAC3 and cathepsins are not clear. We have reported that HDAC3-deficient macrophages have elevated levels of cathepsins and CTSB promoted RIP1 degradation through the lysosomal degradation pathway. We have also shown that HDAC3-deficient macrophages are impaired in NF-kB activation and downregulating numerous inflammatory genes in response to TNFa stimulations. More importantly, we have demonstrated that mice with macrophage specific HDAC3 knockout are more susceptible to infection with bacteria such as *pseudomonas aeruginosa*. Currently, US FDA has approved four HDAC inhibitors (Vorinostat, Romidepsin, Belinostat, and Panobinostat) and Chinese FDA has approved two HDAC inhibitors (Chidamide and HBI-800) for various cancer treatments, while many more HDAC inhibitors are at different stages of clinical trials. In addition, specific inhibitors for individual HDAC family members including proteolysis-targeting chimera (PROTAC) compounds that can mediate degradation of specific HDAC family members are being developed. Our studies uncover the role of HDAC3 in facilitating NF-κB activation and inflammatory responses by preventing cathepsin-mediated RIP1 degradation as well as the requirement of its expression in macrophages for host defense against bacterial infection, which indicates the importance of future work in considering the risk factors of infections in the clinical applications of HDAC inhibitors.

## Materials and methods

### Reagents and plasmids

Antibodies against HDAC3 (#85057, 1:1000 for western blot; #3949, 1:100 for immunofluorescence staining), HDAC1 (#5356, 1:1000), HDAC2 (#5113, 1:1000), HDAC8 (#66042, 1:1000), CTSB (#31718, 1:1000 for western blot; 1:100 for immunofluorescence staining), CTSD (#69854, 1:1000), RIP1 (#3493, 1:1000), GAPDH (#2118, 1:2000), β-ACTIN (#4970, 1:2000), Phosphorylated-RIP1 (#38662, 1:1000), Phosphorylated-P65 (#3033, 1:1000), P65 (#8242, 1:1000 for western blot; 1:100 for immunofluorescence staining), H3K27ac (#8173, 1:50 for CHIPseq) were purchased from Cell Signaling Technology (CST). Leupeptin (#S7380), CA-074 (#S7420) were bought from Selleck. MG132 (#M8699) was bought from Sigma-Aldrich. TNFα (#410-MT-010) was bought from R&D systems. Lipo3000 (InvitroGen, #L3000015) was used for the transfection experiment. PCMV-C-EGFP (#D2626) was bought from Beyotime Biotechnology. The plasmids needed for the experiment were synthesized by GENEWIZ.

### Cell and lenti-virus

RAW264.7 (murine monocytic cell line, ATCC: TIB-71), 293T and Neuro-2a cells were cultured in Dulbecco’s-modified Eagle’s medium (DMEM) (Gibco) containing 10% fetal bovine serum (FBS) (Peak) and 50 units/ml penicillin/streptomycin (Gibco). BMDMs were generated from the bone marrow of 8-week-old mice and cultured in RPMI-1640 medium (Gibco) with 10% FBS and recombinant mouse granulocyte–macrophage colony-stimulating factor (PeproTech, 50 ng/ml).

CRISPR/Cas9 system was used for construct of *Hdac1*^*−/−*^*, Hdac2*^*−/−*^*, Hdac3*^*−/−*^ and *Hdac8*^*−/−*^ RAW264.7 cells. The sequences of small guide RNA (sgRNA) were listed in Additional file 1: Table S1. sgRNA was synthesized and ligased into lenti-V2 vector. And both sgRNA loaded lenti-V2 vector, pMD2.G (Addgene, #12259) and psPAX2 (Addgene, #12260) were then transfected into 293T cells by Lipo3000 to obstain Lentivirus. Lentivirus containing supernatant was collected and used for infecting RAW264.7 cells. After 48 h infection, RAW264.7 cells were then selected with 4 μg/ml puromycin (InvivoGen, #ant-pr-1) in DMEM complete medium for one week. Flow cytometry was used to separate the cells into monoclonal and western blot technology was used to identify whether the gene was knocked out.

### Quantitative real-time PCR (RT-qPCR)

RNA of 2 × 10^6^ cells seeded on six-well plates was extracted by NucleoZol (MNG). Briefly, 600 μl NucleoZol was added with 240 μl ddH_2_O and then centrifugated at 12,000*g*, 4 ℃ for 15 min, and 700  μl supernatant was added with 700  μl isopropanol. After centrifugation, the RNA precipitation was washed by 75% ethanol for two times. PrimeScript™II 1st Strand cDNA Synthesis Kit (Takara, #6210A) was used for Reverse transcription of RNA into complementary DNA (cDNA). And SYBR Green (Takara) was used for qPCR. All the steps are done according to the manufacturer’s instructions. The sequences for primer pairs are listed in Additional file 1: Table S2. RNAseq was done by the company (Novogene) for database construction and sequencing.

### Western blot

Cells were lysed for 30 min on ice by lysis buffer (Beyotime Biotechnology, #P0013) which contained phosphatase inhibitor cocktail (Roche, #04906845001) and complete protease inhibitor cocktail (Roche, #04693132001). After centrifugation at 12,000*g*, 4 ℃ for 15 min and boiled with 4X Protein SDS PAGE Loading (Takara, #9173) for 10 min, the proteins were separated through SDS-PAGE gel and transferred onto PVDF membrane. The membrane was blocked with 5% skimmed milk for 2 h, and then incubated with interested antibodies at 4 ℃ overnight. After stained with HRP-coupled secondary antibodies for 2 h, the proteins on membrane were then detected by Chemiluminescence system (Bio-Rad ChemiDoc MP).

### Chromatin immunoprecipitation (ChIP)

Chromatin IP Kit (CST, #9005) was used for Chromatin Immunoprecipitation. All process was done according to manufacturer’s protocol. In brief, RAW264.7 cells in 20 ml DMEM complete medium were added with 540 μl 37% formaldehyde for 10 min at room temperature and then added with 2 ml 10X glycine for 5 min. After washed by 20 ml cold PBS for 3 times, cells were subject to micrococcal nuclease digestion and sonication through which DNA was processed to the length of approximately 150–900 bp. Chromatin concentration was measured using 10% of the sample after DNA purification. 10 μg chromatin was then mixed with indicated antibodies at 4 ℃ overnight. 30 μl Protein G Magnetic Beads were added to each IP reaction and incubate for 2 h at 4 ℃. After washing in low salt buffer for 3 times and in high salt buffer for 1 time, beads were resuspended with ChIP Elution Buffer. After eluted from beads through vortexing (1200 rpm) at 65 ℃ for 30 min, Chromatin was reverse crosslinked with RNase and proteinase K at 65 ℃ for 2 h. Then DNA solution was then subjected for library establishment after DNA purification.

KAPA HyperPlus Kit (KAPA Biosystems, #KK8514) was used for Library establishment. All process was done according to manufacturer’s protocol. In brief, 50 μl sample was subjected to End Repair and A-tailing at 65 ℃ for 30 min. And then the sample was ligased with different adapter at 20 ℃ for 15 min. The sample was mixed with 88 μl KAPA Pure Beads at room temperature for 15 min and then placed on a magnetic frame. After washed by 80% ethanol for 3 times, DNA was amplified with following PCR program:98 ℃, 45 s; then 8 cycles of 98 ℃, 15 s, 60 ℃, 30 s and 72 ℃, 30 s; 72 ℃, 1 min; 4 ℃, ∞. The sample was again mixed with 88 μl KAPA Pure Beads at room temperature for 15 min and then placed on a magnetic frame. After washed by 80% ethanol for 3 times, DNA was ready for sequencing by company (Novogene).

### Immunofluorescence staining

RAW264.7 cells seeded on Glass Bottom Dish (Cellvis, #D29-10-1.5-N) were fixed using 4% paraformaldehyde for 15 min, permeabilized by 0.1% Triton X-100 (Sigma-Aldrich, #T9284) for 10 min, and blocked with 1% BSA (Beyotime Biotechnology, #ST023) for 1 h at room temperature. They were then stained with anti-CTSB antibodies (CST, #31718, 1:100), anti-HDAC3 antibodies (CST, #3949, 1:100) or anti-P65 antibodies (CST, #8242, 1:100) overnight. After washed by PBS three times, they were followed reacted with Alexa Fluor 488-conjugated anti-mouse IgG secondary antibody (life technologies, #A11001, 1:1000). The nuclei were stained with DAPI (Beyotime Biotechnology, #C1002, 1:1000).

Neuro-2a cells seeded on Glass Bottom Dish were transfected with 1.5 μg PCMV-C-EGFP-RIP1 for 24 h and CA-074 was added at 12 h after transfection. Then the cell culture medium was removed and lyso-tracker (Solarbio, #L8010, 1:15,000) staining solution was added for 1 h at 37 ℃. Cells were washed by PBS for 3 times and The nuclei were stained with Hoechst (Beyotime Biotechnology, #C1027, 1:100).

Cells were imaged on a confocal microscope (Nikon Tl-S).

### Mouse acute lung injury (ALI) model

*Hdac3*^*f/f*^ mice on a C57BL/6 J background were bought from the Jackson Lab (Stock number: 024119). The *Lysm-Cre* mice were bought from the Jackson Laboratory (Stock number: 004781). All mice were bred in Animal center of Suzhou Institute of Systematic Medicine. All animal experiments were approved by the Institutional Animal Care and Use Committee (IACUC) of Suzhou Institute of Systems Medicine (ISM-IACUC-0019-R). Age-matched male *Lysm*^*Cre*^ and *Lysm*^*Cre*^*Hdac3*^*f/f*^ littermates were used for all experiments. In LPS-mediated acute lung injury model, 5 mg/kg LPS was injected into the airway of mice. After 12 h, the mice were executed for BALF extraction. In *pseudomonas aeruginosa*-mediated acute lung injury model, the mice were intratracheal instillation of a dose of *pseudomonas aeruginosa* (2 × 10^7^ CFU/per mouse). The survival rate was observed every 12 h up to 120 h. The temperature of mice was monitered until death appears. BALF extraction and lung tissue collection were done in 36 h after *pseudomonas aeruginosa* infection.

### Bronchoalveolar lavage fluid (BALF) extraction and analysis

An incision in the bronchus of mice was cut with scissors after anesthesia. Then the syringe was used for injecting 1 ml cold PBS into the airway and about 70–80% PBS was withdrawn. After twice the same steps, about 1.5 ml BALF was obstained. Centrifugation was performed to separate the cells and supernatants for inflammatory factors assay.

### Enzyme-linked immunosorbent assay (ELISA)

IL-6 (eBioscience, #85-88-7066-77), TNFα (eBioscience, #85-88-7324-88) and HMGB1 (IBL, #ST51011) were detected with ELISA kits according to the manufacturer’s protocols.

### Histological analysis

The scissors were used to open the thoracic cavity of mice and the syringe was used to collect the blood from heart to avoid blood contamination after anesthesia. Then the lung was resected and fixed in 4% paraformaldehyde (PFA). The samples were then embedded with paraffin and sliced into 4 um sections for haematoxylin and eosin (H&E) staining. The injury score was calculated according to previous research [34].

### Statistical analysis

 Data are presented as mean ± SEM. The significance of difference between groups was detected by two-tailed Student’s t-test or one-way analysis of variance test as appropriate. Mouse survival curve analysis was calculated by the log rank test. GraphPad Prism5 was used for data analysis. *P*-value < 0.05 was considered statistically significant.

## Supplementary Information

**Additional file 1: Figure S1.** Identification of mice of different genotypes. **A** PCR analysis of the expression of *Flox-Hdac3* and *Cre* gene in *Lysm*^*Cre*^*Hdac3*^*f/f*^, *Lysm*^*Cre*^*Hdac3*^*f*/w^, *Lysm*^*Cre*^, *Hdac3*^*f/f*^, *Hdac3*^*f*/*w*^ and WT mice. **B** Western blot analysis of HDAC3 of BMDMs from *Lysm*^*Cre*^ and *Lysm*^*Cre*^*Hdac3*^*f*/*f*^ mice. **Figure S2.** HDAC3 deficient macrophages have elevated expression of *Ctsd*. **A **PCR analysis of *Ctsd *in Vector, *Hdac1*^*−/−*^, *Hdac2*^*−/−*^, *Hdac3*^*−/−*^,* Hdac8*^*−/−*^ RAW264.7 cells. **B-C** Western blot analysis of CTSD in Vector, *Hdac1*^*−/−*^, *Hdac2*^*−/−*^, *Hdac3*^*−/−*^,* Hdac8*^*−/−*^ RAW264.7 cells. Data are representative of three independent experiments and showed as mean ± SEM. *P < 0.05 and **P < 0.01. **Figure S3.** HDAC3 is mainly located in the nucleus. Confocal micrographs of RAW264.7 stained with DAPI (blue, DNA) and antibodies against HDAC3 (AF488). Scale bar: 100/10 μm. Figures are representative of three independent experiments. **Figure S4.** RIP1 is degraded in lysosomes. Confocal micrographs of Neuro-2a transfected with PCMV-C-EGFP-RIP1 and stained with Hoechst (blue, DNA) and Lyso-Tracker Red after CA-074 (10 μM) stimulation for 12 h. Figures are representative of three independent experiments. **Table S1.** sgRNA used for the construction of knockout cell lines. **Table S2.** Primers used for polymerase chain reaction in this paper.

## Data Availability

The datasets used and/or analyzed during the current study are available from the corresponding author on reasonable request. RNA-seq original data have been submitted to the Gene Expression Omnibus (GEO) database (GSE197989). CHIP-seq original data have been submitted to the Gene Expression Omnibus (GEO) database (GSE198218).
