# Supplementary material for: Histone deacetylase 3 facilitates TNFα-mediated NF-κB activation through suppressing CTSB induced RIP1 degradation and is required for host defense against bacterial infection
Source: Cell Biosci. 2022 Jun 3;12:81. doi: 10.1186/s13578-022-00814-6 (PMC9164478; doi:10.1186/s13578-022-00814-6)
Supplement: Supplementary file 1 — Additional file 1: Figure S1. Identification of mice of different genotypes. A PCR analysis of the expression of Flox-Hdac3 and Cre gene in LysmCreHdac3f/f, LysmCreHdac3f/w, LysmCre, Hdac3f/f, Hdac3f/w and WT mice. B Western blot analysis of HDAC3 of BMDMs from LysmCre and LysmCreHdac3f/f mice. Figure S2. HDAC3 deficient macrophages have elevated expression of Ctsd. A PCR analysis of Ctsd in Vector, Hdac1−/−, Hdac2−/−, Hdac3−/−, Hdac8−/− RAW264.7 cells. B-C Western blot analysis of CTSD in Vector, Hdac1−/−, Hdac2−/−, Hdac3−/−, Hdac8−/− RAW264.7 cells. Data are representative of three independent experiments and showed as mean ± SEM. *P < 0.05 and **P < 0.01. Figure S3. HDAC3 is mainly located in the nucleus. Confocal micrographs of RAW264.7 stained with DAPI (blue, DNA) and antibodies against HDAC3 (AF488). Scale bar: 100/10 μm. Figures are representative of three independent experiments. Figure S4. RIP1 is degraded in lysosomes. Confocal micrographs of Neuro-2a transfected with PCMV-C-EGFP-RIP1 and stained with Hoechst (blue, DNA) and Lyso-Tracker Red after CA-074 (10 μM) stimulation for 12 h. Figures are representative of three independent experiments. Table S1. sgRNA used for the construction of knockout cell lines. Table S2. Primers used for polymerase chain reaction in this paper. [file 13578_2022_814_MOESM1_ESM.pdf]

Histone deacetylase 3 facilitates TNFα-mediated NF-κB activation through suppressing CTSB induced RIP1 degradation and is required for host defense against bacterial infection

Liping Yang[1, 2, 3*], Shengchuan Chen[2, 3, 4*], Jingyan Xia[5*], Ying Zhou[2, 3, 6], Linan Peng[2, 3, 7], Huimin Fan[2, 3], Yu Han[1, 2, 3], Lihua Duan[7†], Genhong Cheng[8†], Heng Yang[2, 3†], Feng Xu[1†]

1 Department of Infectious Diseases, The Second Affiliated Hospital, Zhejiang University School of Medicine, Hangzhou, 310009, China

2 Institute of Systems Medicine, Chinese Academy of Medical Sciences & Peking Union Medical College, Beijing, 100005, China

3 Suzhou Institute of Systems Medicine, Suzhou, 215123, China

4 Department of Gastrointestinal Surgery, The First Affiliated Hospital, Zhejiang University School of Medicine, Hangzhou, 310009, China

5 Department of Radiation Oncology, The Second Affiliated Hospital, Zhejiang University School of Medicine, Hangzhou, 310009, China.

6 Department of Obstetrics and Gynecology, The First Affiliated Hospital of Soochow University, Suzhou, 215123, China

7 Department of Rheumatology and Clinical Immunology, Jiangxi Provincial People's Hospital Affiliated to Nanchang University, Nanchang, 330000, China.

8 Department of Microbiology, Immunology and Molecular Genetics, University of California Los Angeles, CA, 90095, USA.

* L.Y., S.C. and J.X. share the co-first authorship

† F.X., H.Y., G.C. and L.D. are co-corresponding authors.

Lead contact: Feng Xu, xufeng99@zju.edu.cn.

This file includes

Fig. S1-S4

Table S1-S2

Figure S1

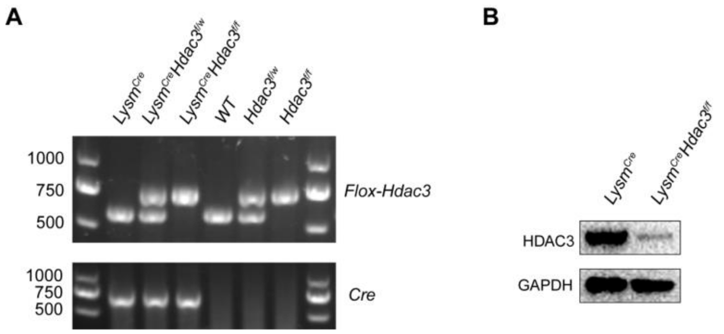

Fig. S1. Identification of mice of different genotypes.

A PCR analysis of the expression of *Flox-Hdac3* and *Cre* gene in $Lysm^{Cre}Hdac3^{f/f}$, $Lysm^{Cre}Hdac3^{f/w}$, $Lysm^{Cre}$, $Hdac3^{f/f}$, $Hdac3^{f/w}$ and WT mice. **B** Western blot analysis of HDAC3 of BMDMs from $Lysm^{Cre}$ and $Lysm^{Cre}Hdac3^{f/f}$ mice.

Figure S2

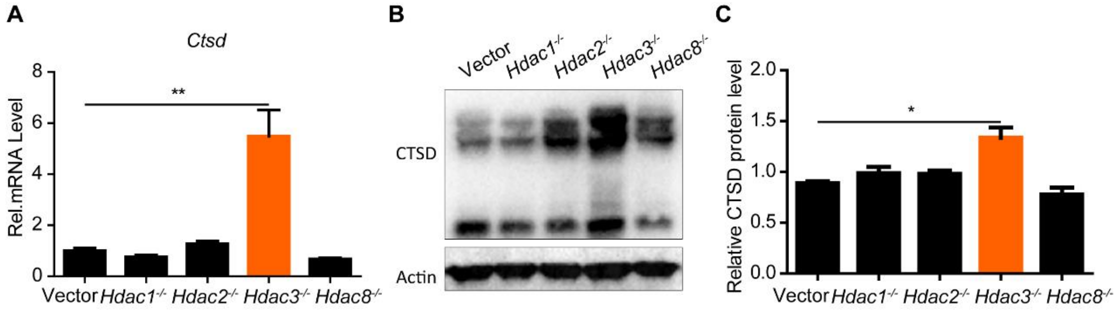

Fig. S2. HDAC3 deficient macrophages have elevated expression of *Ctsd*.

A PCR analysis of *Ctsd* in Vector, $Hdac1^{-/-}$, $Hdac2^{-/-}$, $Hdac3^{-/-}$, $Hdac8^{-/-}$ RAW264.7 cells. **B-C** Western blot analysis of CTSD in Vector, $Hdac1^{-/-}$, $Hdac2^{-/-}$, $Hdac3^{-/-}$, $Hdac8^{-/-}$ RAW264.7 cells. Data are representative of three independent experiments and showed as mean ± SEM. *$P<0.05$ and **$P<0.01$.

Figure S3

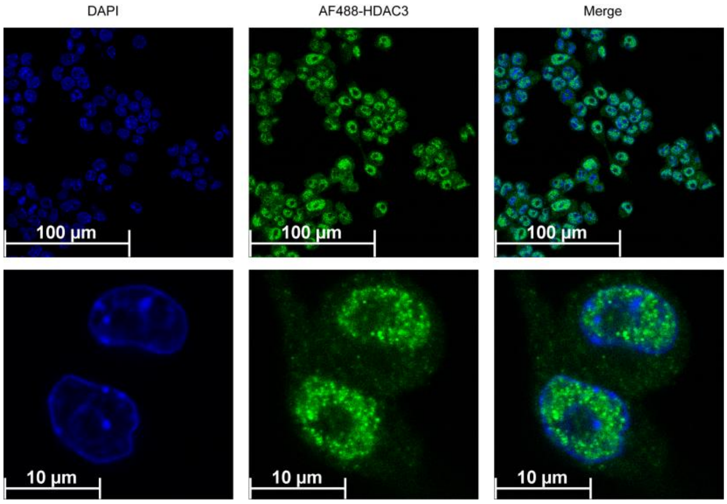

Fig. S3. HDAC3 is mainly located in the nucleus.

Confocal micrographs of RAW264.7 stained with DAPI (blue, DNA) and antibodies against HDAC3 (AF488). Scale bar: 100/10 μm. Figures are representative of three independent experiments.

Figure S4

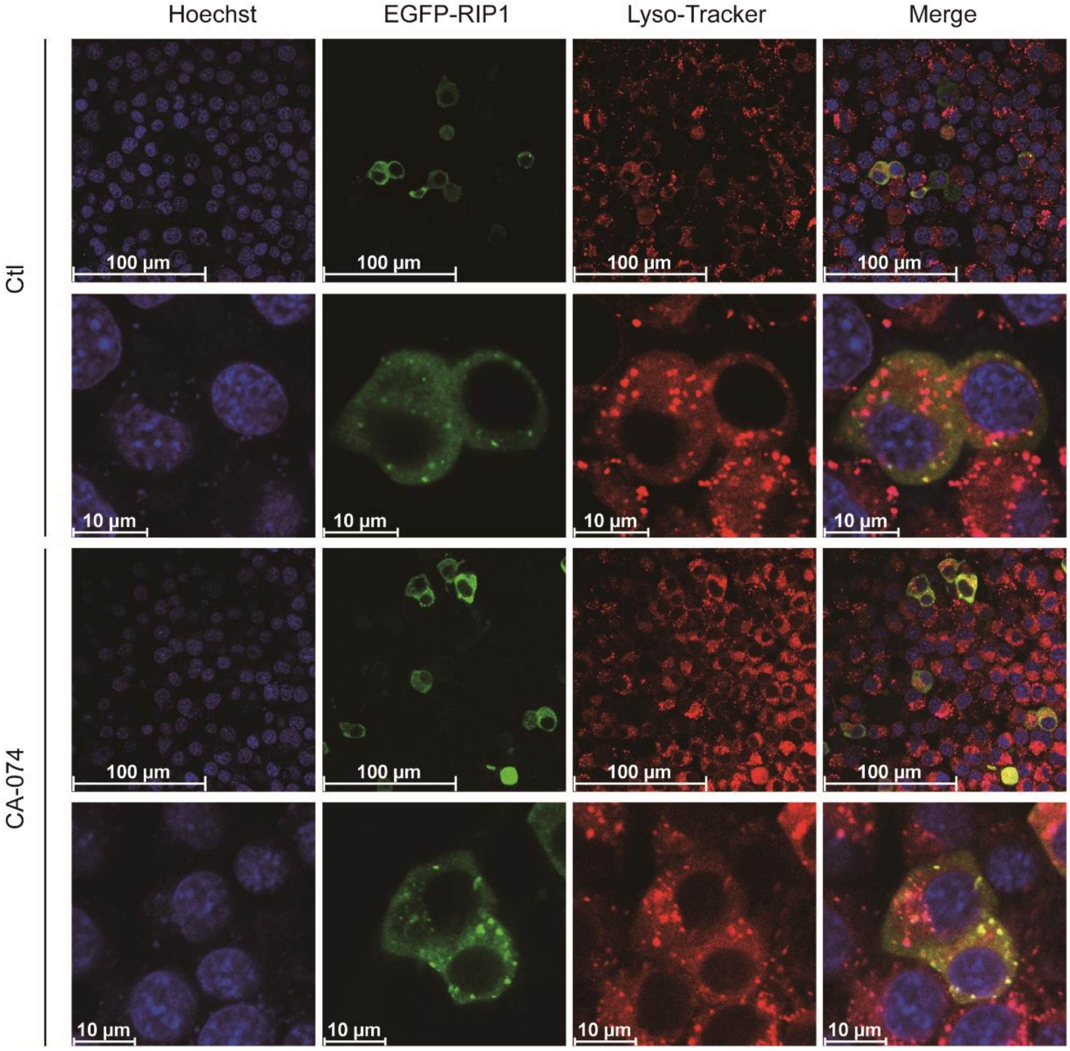

Fig. S4. RIP1 is degraded in lysosomes.

Confocal micrographs of Neuro-2a transfected with PCMV-C-EGFP-RIP1 and stained with Hoechst (blue, DNA) and Lyso-Tracker Red after CA-074 (10 μM) stimulation for 12 h. Figures are representative of three independent experiments.

Table S1. sgRNA used for the construction of knockout cell lines.

sgRNA	Sequences
Mouse HDAC1 sgRNA-F	CACCG CTTTCAAGTCAATGTTGGTG
Mouse HDAC1 sgRNA-R	AAAC CACCAACATTGACTTGAAAG C
Mouse HDAC2 sgRNA-F	CACCG CCATGAAGCCTCATAGAATC
Mouse HDAC2 sgRNA-R	AAAC GATTCTATGAGGCTTCATGG C
Mouse HDAC3 sgRNA-F	CACCG CCCAATGAAACCTCATCGCC
Mouse HDAC3 sgRNA-R	AAAC GGCGATGAGGTTTCATTGGG C
Mouse HDAC8 sgRNA-F	CACCG GATGGTCCTCATCACCTTCT
Mouse HDAC8 sgRNA-R	AAAC AGAAGGTGATGAGGACCATC C

Table S2. Primers used for Polymerase Chain Reaction in this paper.

Primers	Sequences
Mouse IL-1β forward primer	GAAATGCCACCTTTTGACAGTG
Mouse IL-1β reverse primer	TGGATGCTCTCATCAGGACAG
Mouse Mcp1 forward primer	TTAAAAACCTGGATCGGAACCAA
Mouse Mcp1 reverse primer	GCATTAGCTTCAGATTTACGGGT
Mouse Mip2 forward primer	CCAACCACCAGGCTACAGG
Mouse Mip2 reverse primer	GCGTCACACTCAAGCTCTG
Mouse Cox2 forward primer	TTCAACACACTCTATCACTGGC
Mouse Cox2 reverse primer	AGAAGCGTTTGCGGTACTCAT
Mouse β-ACTIN forward primer	GGCTGTATTCCCCTCCATCG
Mouse β-ACTIN reverse primer	CCAGTTGGTAACAATGCCATGT
Mouse Ctsa forward primer	CCCTCTTTCCGGCAATACTCC
Mouse Ctsa reverse primer	CGGGGCTGTTCTTTGGGTC
Mouse Ctsb forward primer	TCCTTGATCCTTCTTTCTTGCC
Mouse Ctsb reverse primer	ACAGTGCCACACAGCTTCTTC
Mouse Ctsc forward primer	CAACTGCACCTACCCTGATCT
Mouse Ctsc reverse primer	TAAAATGCCCGGAATTGCCCA
Mouse Ctsd forward primer	GCTTCCGGTCTTTGACAACCT
Mouse Ctsd reverse primer	CACCAAGCATTAGTTCTCCTCC
Mouse Ctse forward primer	GACATCAGTCCCTTCGGAAGA
Mouse Ctse reverse primer	AGGGGTTCATTGACACTCGAATA
Mouse Ctsf forward primer	CCCTGGAAGCCACACTAGAG
Mouse Ctsf reverse primer	GGGCTACAGTCCCTCCTCAG
Mouse Ctsg forward primer	AGGGTTTCTGGTGCGAGAAG
Mouse Ctsg reverse primer	GTTCTGCGGATTGTAATCAGGAT
Mouse Ctsh forward primer	ACCGTGAACGCCATAGAAAAG
Mouse Ctsh reverse primer	TGAGCAATTCTGAGGCTCTGA
Mouse Ctsk forward primer	GAAGAAGACTCACCAGAAGCAG
Mouse Ctsk reverse primer	TCCAGGTTATGGGCAGAGATT
Mouse Ctsl forward primer	ATCAAACCTTTAGTGCAGAGTGG

Mouse Ctsl reverse primer	CTGTATTCCCCGTTGTGTAGC
Mouse Ctso forward primer	CAGCGTGGTGAGTGCCATAG
Mouse Ctso reverse primer	ACCGAGGCAGCCAGAATTATTA
Mouse Ctss forward primer	CCATTGGGATCTCTGGAAGAAAA
Mouse Ctss reverse primer	TCATGCCCACTTGGTAGGTAT
Mouse Ctsw forward primer	TGACTCCCTCCTCACCAAGG
Mouse Ctsw reverse primer	GCTGGGTTCCAGTAACTCCG
Mouse Ctsz forward primer	GGCCAGACTTGCTACCATCC
Mouse Ctsz reverse primer	ACACCGTTCACATTTCTCCAG
